# Health Burdens and SES in Alabama: Using Geographic Information System to Examine Prostate Cancer Health Disparity

**DOI:** 10.3390/cancers14194824

**Published:** 2022-10-02

**Authors:** Seela Aladuwaka, Ram Alagan, Rajesh Singh, Manoj Mishra

**Affiliations:** 1 Cancer Biology Research and Training, Alabama State University, Montgomery, AL 36104, USA; 2 Department of Advancement Studies, Alabama State University, Montgomery, AL 36104, USA; 3 Department of Microbiology, Biochemistry & Immunology and Cancer Health Equity Institute, Morehouse School of Medicine, Atlanta, GA 30310, USA; 4 Department of Biological Sciences, Alabama State University, Montgomery, AL 36104, USA

**Keywords:** geographic information system, prostate cancer, SES, health disparities, obesity, diabetes

## Abstract

**Simple Summary:**

The focus of the research was to examine the relationship between Socioeconomic status and prostate cancer in Alabama’s Black Belt region. The cancer rate in Alabama is high, and the state has one of the highest rates of prostate cancer in the USA. The research aims to identify probable reasons, raise awareness, and propose cancer prevention policies. The Geographic Information System, a robust technology, has been adopted to understand Alabama’s county-level prostate cancer incidence and mortality and its association with socioeconomic and health disparities. The analysis indicated an apparent socioeconomic disparity between the Black Belt and Non-Black Belt counties of Alabama. The poverty rate is higher in Black Belt counties. The data revealed that the preexisting condition of diabetes and obesity is closely associated with prostate cancer. Also, incidence and mortality disparities strongly relate to socioeconomic status, and the preexisting condition of obesity and diabetes adds to prostate cancer incidences. Poverty is the root course of inequalities in education, income, and healthcare facilities, particularly among African Americans, contributing to Alabama’s health burden of prostate cancer. The study proposes effective health policy intervention to prevent and reduce prostate cases and mortality among underserved communities in Alabama.

**Abstract:**

Socioeconomic disparities influence the risk of many diseases, including cancer. The cancer rate in Alabama is high, and the state has one of the highest rates of prostate cancer in the USA. Alabama’s counties are embedded with socioeconomic disparities, politics, race, ethnicity, and oppression, among which social equity and socioeconomic status (SES) been closely associated with prostate cancer. The Geographic Information System (GIS) has become a valuable technology in understanding public health in many applications, including cancer. This study integrates Alabama’s county-level prostate cancer incidence and mortality and its association with socioeconomic and health disparities. We conducted robust data mining from several data sources such as the Alabama State Cancer Profile data, Alabama Department of Health, American Cancer Society, Center for Disease Control, and National Cancer Institute. The research method is the Geographic Information System (GIS), and we employed prostate cancer data within GIS to understand Alabama’s prostate cancer prevalence regarding SES. The GIS analysis indicated an apparent socioeconomic disparity between the Black Belt and Non-Black Belt counties of Alabama. The Black Belt counties’ poverty rate is also remarkably higher than non-Black Belt counties. In addition, we analyzed the median household income by race. Our analysis demonstrates that the Asian background population in the state earned the highest median income compared to non-Hispanic whites and the African American population. Furthermore, the data revealed that the preexisting condition of diabetes and obesity is closely associated with prostate cancer. The GIS analysis suggests that prostate cancer incidence and mortality disparities are strongly related to SES. In addition, the preexisting condition of obesity and diabetes adds to prostate cancer incidences. Poverty also reflects inequalities in education, income, and healthcare facilities, particularly among African Americans, contributing to Alabama’s health burden of prostate cancer.

## 1. Introduction 

Evaluating the impact of SES on prostate cancer and mapping the spatial distribution is essential to understand the health disparity and health equity measures. Prostate cancer is one of the most significant health threats in the USA, with the highest incidence in African American men possessing the highest death rate and shortest survival rate of any racial/ethnic group in the U.S. [1,2]. Alabama is one of the leading states in the nation in the cancer epidemic. About one in nine men are diagnosed with prostate cancer during their lifetime [3,4]. The incidence rate of prostate cancer in the United States is a growing burden due to several factors, including socioeconomic factors, cultural differences, and health disparities. Socioeconomic inequalities result in unequal access to opportunities and resources, such as work, wealth, income, education, housing, healthy food, and overall standard of living. The combination of issues underlines the severity of cancer prevalence worldwide and the need for public health education and health equity extension. In the U.S., the cancer incidence rate is 442.4 per 100,000 men and women per year [2,5,6]. The cancer death rate (cancer mortality) is estimated to be 158.3 per 100,000 men and women per year [5,6]. At the same time, 1 in 10 among the non-Hispanic male population is likely to develop prostate cancer [1,7,8]. The data also reveal that African American males are more likely to die from prostate cancer than non-Hispanic, with mortality rates of 1 in 25 and 1 in 45, respectively [1,7,8,9]. Recent data indicate that prostate cancer has been the highest estimated cancer rate in African American males, making up 30% of all demographics [3,10,11,12]. Interesting, prostate cancer was the second highest among African American men in estimated death rates [3,12]. On the other hand, prostate cancer is one of the most common cancers among Alabama men [2]. Therefore, in this article, we used GIS to demonstrate the impact of SES on prostate cancer incidence in the black-belt counties of Alabama.

## 2. Methodology

Geographic Information Systems can illustrate geospatial inequalities in development programs and underline health disparity’s impact on minority communities in Alabama. This research employs GIS methodology to incorporate county-level prostate cancer data and explore the correlation between prostate cancer prevalence and socioeconomic factors. The advancements in GIS (hardware and software) technology, coupled with spatiotemporal and methodological enhancements and the widespread accessibility of geographically referenced spatial and statistical data, have led to general use in cancer research.

Although multiple underpinnings cause prostate cancer incidences in Alabama, this research explores the impact of socioeconomic determinants, including preexisting conditions such as diabetes and obesity, on health disparity. SES is defined by an individual or group’s social standing or class and is generally measured using education, income, and occupation [13,14,15,16,17]. Analysis of the SES helps understand inequities in access to resources and issues related to privilege, power, and control. SES directly impacts demographics’ Social Determinants of Health (SDH), such as poverty, unequal healthcare access, lack of education, stigma, and racism [13]. SDH is the non-medical factor influencing health outcomes [18,19,20,21,22,23]. These include the conditions in which people are born, grow, work, live, and age and the broader set of forces and systems shaping the needs of daily life [22]. According to the U.S. Department of Health and Human Services, several indicators measure aspects of SDH, including economic stability, education, general well-being, access to health care, housing quality, food quality, and the conditions of the surrounding natural environment.

Therefore, this study focuses on the Black Belt regions in Alabama. The Black Belt region has some of the highest health disparities in the state due to a lack of access to essential economic and social resources leading to poor health among African American populations in Alabama. Alabama’s Black Belt region consists of a distinct social and cultural boundary, and it extends from Virginia to Texas along with the Southeastern USA. It is mainly affected by poverty, limited healthcare systems, a lack of economic prospects, and poor access to healthy lifestyle selections. The Black Belt region is home to a large African American population. Most counties have a high percentage African American population.

### Prostate Cancer and SES

Numerous data sources have explored the relationship between prostate cancer and SES. The geospatial correlation between cancer and socioeconomic stress emphasizes Alabama’s health disparity and equity. A combination of socioeconomic factors and state and federal-level cancer epidemic data sources have also underscored high prostate cancer prevalence in Alabama’s Black Belt region (predominantly African American). Some of these data sources are (1) the Alabama Public Health Department, (2) the Alabama Statewide Cancer Registry, (3) the Alabama Data and Statistical Atlas, and (4) the National Cancer Institute. We also used secondary data, such as poverty and education data from the Alabama Possible [24] and the U.S. Census [25], national and state-level cancer, and socioeconomic (e.g., poverty, household income, obesity, diabetes, race, and cancer statistics) databases. The socioeconomic and prostate cancer statuses were assembled in Alabama’s 67 counties. We examined the prostate cancer status and its association with socioeconomic information using GIS technology. Furthermore, statistical analysis was performed to identify the spatial relationships between prostate cancer, SES, and, most importantly, the health disparities between counties. Finally, the GIS analysis also demonstrated that effective cancer prevention programs are needed to eliminate health disparities and enhance health equity among underserved and rural populations of Alabama.

## 3. Results and Discussion

**Figure 1:**
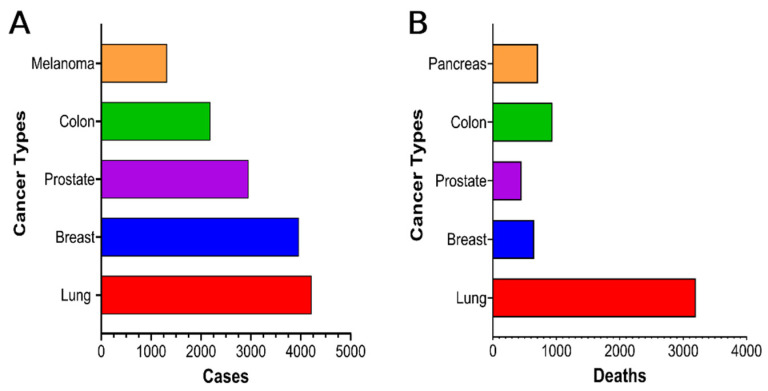
The top five cancer incidences in Alabama. (**A**) shows Alabama’s top five cancer cases, while (**B**) indicates Alabama’s top five cancer deaths. Prostate cancer is a health disparity disease. It is among the top five in both categories (cases and deaths). Data source: Alabama Statewide Cancer Registry, Alabama Department of Public Health (2020) [2], accessed on 12 February 2022.

Alabama is one of the nation’s leading states in the cancer epidemic, including prostate cancer [2]. The unique characteristics of prostate cancer are the distinct racial/ethnic disparities in incidence and mortality rates [26], with the highest rates among African Americans. Out of several cancers, prostate cancer incidence reached the top three and top five in deaths in health epidemics in Alabama (Figure 1A,B). Although several factors influence cancer status, early screening is one of the critical factors that would help cancer reduction [27,28]. It has been demonstrated that early screening reduces the difference in prostate cancer risk by SES [10,26,28,29]. The SES indicators were educational level, income, and homeownership status [26]. According to these findings, higher SES was associated with a higher incidence of low-to-moderate risk prostate cancer but a lower risk of advanced prostate cancer [26]. Higher education was correlated with significantly lower prostate cancer mortality in both control and screening [10,26,27]. There were a reported 4060 prostate cancer cases and approximately 510 prostate cancer mortalities in Alabama [30]. Alabama’s prostate cancer incidence rate is significantly higher than the U.S. rate in all categories (Table 1), including white and black [3,31].

**Figure 2:**
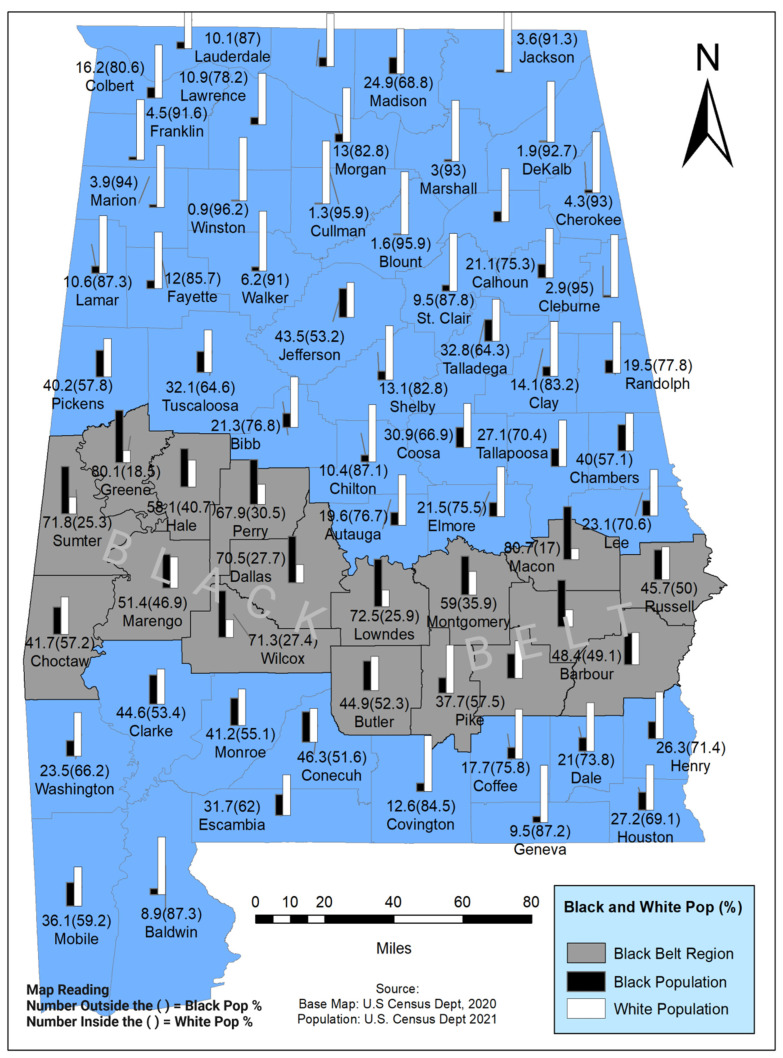
The current population of whites and African Americans (A.A.), as well as their distribution in Alabama. The A.A. population is more concentrated in the Black Belt region (53%), while the statewide A.A. population is 27%. Historically, the SES is compromised in the Black Belt region compared to the non-Black Belt region. This helps understand the impact of SES on prostate cancer health disparity. Data source: U.S. Base Map; U.S. Department of Census (2020) [25], accessed on 25 December 2021.

Seventeen of Alabama’s Black Belt counties are depicted in the Geographic Information Systems (GIS) displays, including white and African American populations (Figure 2). Apart from several metropolitan areas, 41% are rural. Notably, Black Belt counties have the highest percentage of the rural population, where health-related services and transportation are limited and characterized by low SES. Combining the history of economic struggle immensely increases African American communities’ prostate cancer vulnerability in the region. With high poverty, Alabama is one of the nation’s most disadvantaged states regarding its economic development, and the nation’s fifth most impoverished state [24]. More than 800,000 Alabamians, including 256,000 children, live below the federal poverty threshold of USD 25,701 for a family of four. Alabama’s Black Belt region is unique in its cultural, socioeconomic, historical, and political characteristics, with vibrant social and cultural backgrounds. A national survey conducted on trends and patterns of disparity in health-related mortality among U.S. counties between 1980 and 2016 showed large health disparity clusters in the southern belt, including the Black Belt in Alabama. Along with high prostate cancer cases, this region is also exceptionally high in cardiovascular disease, obesity, and diabetes.

According to the Alabama Statewide Cancer Registry (2021), the African American male population faces more cancer incidence (562.4 per 100,000) and mortality (265.9 per 100,000) than the white population cancer incidence (517.8 per 100,000) and mortality (221.6 per 100,000) by a significant amount (Figure 2). This study examines the impact of social-economic status on prostate cancer incidence in Alabama’s counties, particularly in the Black Belt region. 

### 3.1. Geographic Information Systems and Cancer Health Care

Spatial inequality of healthcare development is one of the fundamental issues lacking in minority communities, and it requires broader discussion and policy formulation [32,33,34,35]. The GIS has been employed in numerous physical, social, and environmental research activities. The spatial analysis nature of the GIS has broad potential in public health and medical research. General health issues and cancer epidemics are distributed in the geospatial location, distance, and direction in each state, country, township, and ethnicity. Exploring the combination of social and cancer information in the spatial context (GIS) will help researchers to better understand the ongoing healthcare challenges and help find sustainable solutions [36]. The GIS provides spatial analysis and explains the changing spatial distribution of healthcare, examining its relationship to health outcomes and exploring how healthcare delivery can be improved [37]. 

The unique nature of spatial analysis through GIS provides a set of mechanisms for representing, organizing, exploring, and interpreting the existing spatial distribution of healthcare systems [38]. Examining these relationships is the first step in improving the fight against health disparity. GIS provides decision-makers and healthcare institutions with various innovative perceptions and options. GIS also provides critical support in planning when, where, and how to advance the quality of care, improve the accessibility of service, locate more cost-effective delivery methods, and diversify data accessibility. This study provides valuable spatial recognition of prostate cancer in Alabama using the GIS application, which will offer future solutions to reduce prostate cancer incidence and mortality in Alabama.

### 3.2. Poverty, SES, and Prostate Cancer 

**Figure 3:**
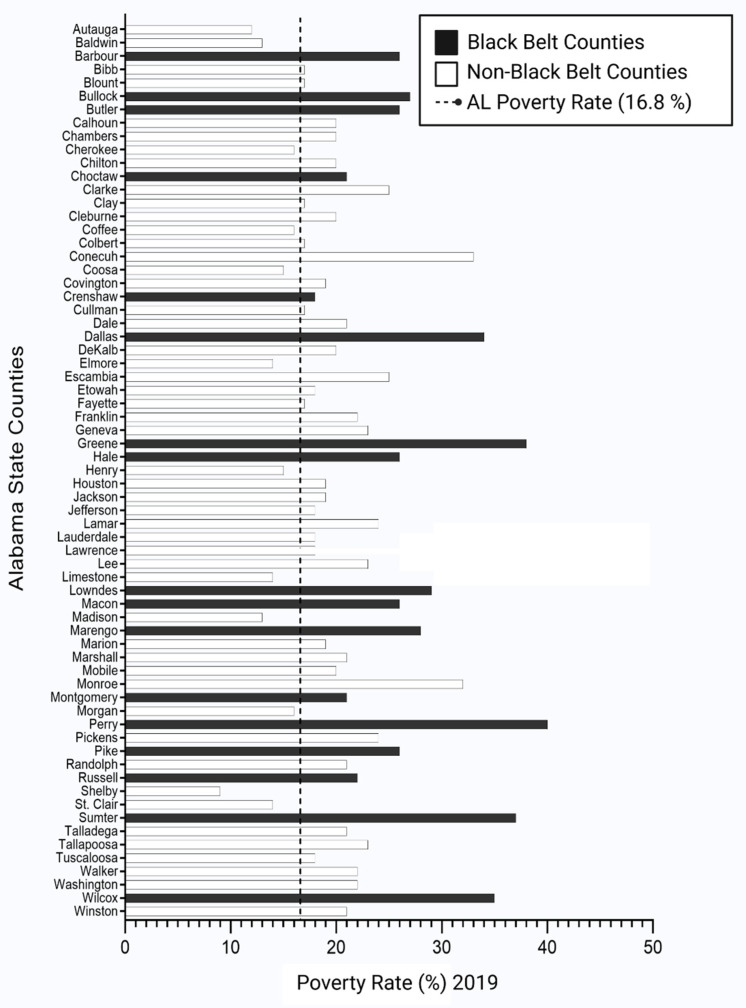
The poverty rate of each county in Alabama (2021). The state-level poverty rate is 16.8 percent (2021). Out of 67 counties in Alabama, 17 are considered Black Belt counties, where most of the population is African American communities. The socioeconomic disparity is so apparent that all 17 Black Belt counties have a higher poverty rate than the state poverty rate. Most importantly, the top 10 poverty counties in the state are recognized from the Black Belt region. The deprived SES enormously has influenced the health status of the people. Data source: U.S. Department of Census (2021) [25], accessed on 30 December 2021.

A comprehensive analysis of different aspects of SES and their relationship with prostate cancer cases in Alabama revealed many underlining factors, such as poverty, education, median household income, the association between diabetes and obesity, and access to health care for prostate cancer. Poverty is a critical issue that determines SES and adversely impacts human health [14,39,40,41,42]. Alabama is the nation’s fifth most impoverished state, and its high poverty rate is concentrated in the Black Belt region (Figure 2). According to data from the Alabama Department of Labor and the U.S. Census, many African American families in Alabama, particularly in the Black Belt counties, have income below the national poverty line (Figure 3). High incidences of poverty link to low SES, inducing a persistent struggle against rising costs of living and necessary but unobtainable medical expenses. The distribution of poverty status in Alabama state is shown in Figure 3. Out of 67 counties, 17 counties in Alabama are considered Black Belt counties. Among the 17 Black Belt counties, 12 counties are the top poverty spots in the state, which illustrates the profound disparity of socioeconomic among minority populations (Figure 3).

**Figure 4:**
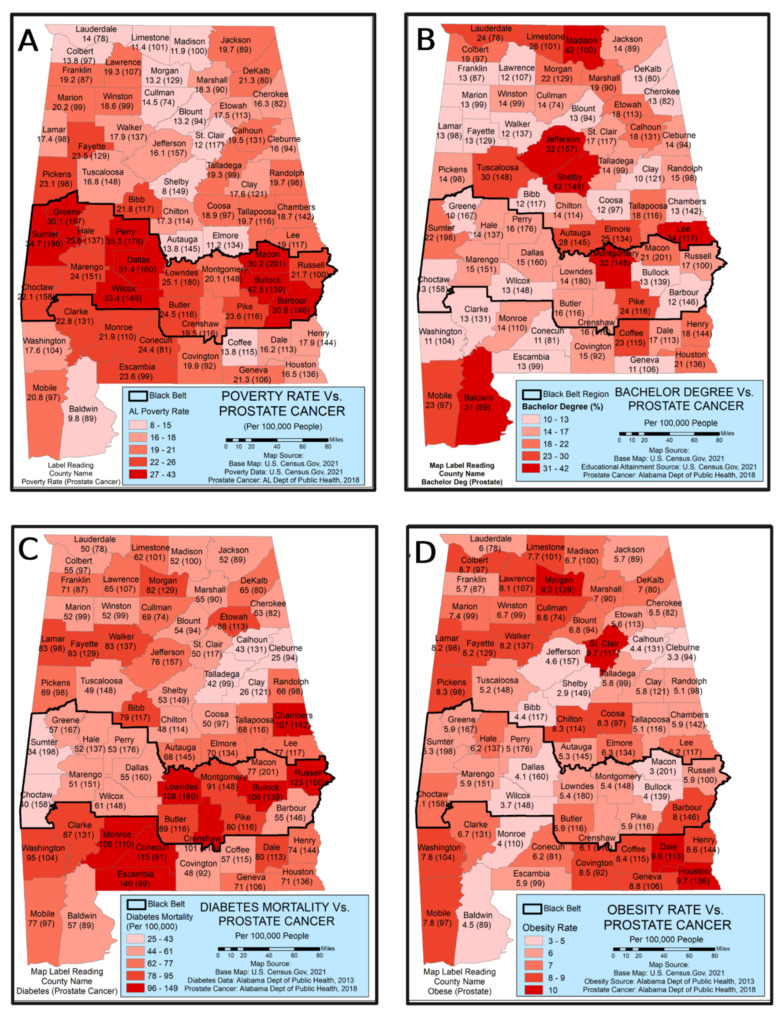
Four causal factors that impact the prostate cancer status in Alabama. This figure illustrates the county-level poverty distribution. (**A**) Bachelor’s degree attainment (**B**), diabetes epidemic (**C**), and obesity status (**D**) in Alabama. These four factors are highly prominent in the black belt region. The poverty rate spread out across the state; the Black Belt counties (dark shaded region) face the highest poverty status compared to non-Black Belt counties. Similarly, there is a significant reduction in education in Black Belt counties with increased diabetes and obesity. Data source: Base Map; U.S. Department of Census (2021), Educational Attainment; U.S. Department of Census (2021), Prostate Cancer Data; Alabama Department of Public Health (2018) [24,25,43], accessed on 20 December 2021.

We conducted a correlation analysis to understand the relationship between poverty and prostate cancer. We collected the prostate cancer and poverty data containing a strong correlation in the Black Belt region in Alabama from the Alabama Department of Public Health. Eight out of seventeen Black Belt counties (Sumter, Green, Perry, Dallas, Wilcox, Macon, Bullock, and Barbour) had a poverty rate above 30 percent. The national poverty level ranges from 11 percent to 13.7 percent, whereas it increased to 16.8 percent in Alabama by 2019. It has been demonstrated that Black Belt counties were below the county’s statewide poverty line compared to other counties across the state of Alabama, and 12 out of 17 counties were among the top 25 percent of most poverty-driven counties in the United States (Figure 3). Compared with the high poverty rate, most Black Belt countries’ prostate cancer numbers are high. Counties such as Sumter (198), Green (167), Perry (176), Dallas (160), Macon (201), Lowndes (180), and Choctaw (158) have high rates of prostate cancer, especially compared with non-Black Belt counties (Figure 4D).

#### Poverty and Prostate Cancer

According to the GIS analysis Sumter, Green, Perry, Dallas, Wilcox, Macon, Bullock, and Barbour counties have well over 150–200 prostate cases per 100,000 people (Figure 4A). The poverty rate of these eight counties is also crucial, with 30 percent and above compared to non-Black Belt counties, with less than 150 cases of prostate cancer and a certainly lower poverty rate (10–20%). Due to its poor socioeconomic condition, the Black Belt region faces an alarming situation regarding better health care. The GIS analysis showed that poverty is associated with prostate cancer, particularly in the Black Belt region. Most of the population in this region is African American, who already face inadequate socioeconomic advancement and affordability in a systemically biased healthcare system. It intensifies the health disparity even more among the minority population in Alabama. 

Prior data found that poverty strongly relates to prostate cancer because it affects every segment of people’s health, suggesting that poverty correlates with an increased risk of prostate cancer regardless of race [42,44]. The level of poverty is also closely related among the black male population prostate cancer rate and increases the likelihood of contracting high-risk diseases [45]. Prostate cancer incidence rates tend to be positively correlated with low-income limitations [29]. The GIS analysis demonstrated that the SES in Black Belt counties arises behind access to health care, transportation, purchasing power on medical supplies, and affordability to health.

### 3.3. Education and Prostate Cancer 

Education is one of the socioeconomic measurements associated with increased risks of prostate cancer and other health issues [46]. This, in turn, also impacts a person’s ability to gain access to health facilities and health insurance. The spatial distribution of percentages of bachelor’s degrees and prostate cancer cases per 100,000 people for each county in Alabama is shown in Figure 4B. The map illustrates that the higher the educational accomplishment, the lower the risk of prostate cancer cases. Some of the counties with the lowest prostate cases include Mobile (23/97), Montgomery (32/148), Lee (34/117), Jefferson (42/149), Lauderdale (24/78), and Madison (42/100). These counties with higher bachelor’s degrees house Alabama’s major university institutions. On the other hand, counties with limited high educational facilities and lower academic degree attainment are associated with higher prostate cancer cases per 100,000 people. Black Belt counties such as Green (10/167), Choctaw (13/158), Lowndes (14/180), Wilcox (13/148, Dallas (15/160), Perry (16/176), and Barbour (12/146) all follow this trend. However, better educational facilities and access to health awareness programs are limited in the Black Belt counties. Counties with predominantly white populations such as Franklin (13/87), Dekalb (13/80), Blount (13/94), Lamar (13/98), Coosa (12/97), and Escambia (13/99) are not nearly as extreme as the previous set. The GIS analysis clearly showed that the two different spatial distributions of prostate cancer cases underscore African American males have more cases than white males, even in similar socioeconomic areas. 

Overall, spatial data on educational attainment and prostate cancer are correlated. The school systems in low-SES communities are often under-resourced, negatively affecting students’ academic progress and outcomes [42,47]. SES affects income and educational attainment, financial security, and subjective perceptions of social status and social class [48]. Prior research revealed that the U.S. mass public education system does not work equally [49]. Those with poor academic performance might face decreasing upward mobility and stunted health status, both currently and later in life [49].

### 3.4. Diabetes, Prostate Cancer, and SES

Diabetes is another closely related preexisting health condition that could influence prostate cancer. Prostate cancer and diabetes are two of the most common chronic diseases that trouble the aging male population [50]. Different epidemiological findings have found a consistent relationship between diabetes and prostate cancer risk [51,52,53]. The CDC (2021) study reported that the incidence of diabetes is higher in the black male population than in other ethnic groups in the United States (Table 2). The black male population’s diabetes level is 13.4 percent, the non-Hispanic white male population has 8.7, and the non-Hispanic black/non-Hispanic white population underscores 1.5 percent cases. Although the preexisting condition of diabetes is closely associated with prostate cancer, the geographic location of the residence is another critical factor that should be considered for cancer incidence. The Black Belt region’s limited socioeconomic development increases the likelihood of contracting conditions such as diabetes, accumulating the effects of several health epidemics in a multiplicative context. 

The CDC (2021) surveyed on death rates vs. cases of diabetes, underlining that non-Hispanic black males represent the highest percentage of deaths with 47.6%, while non-Hispanic white males represent the second highest with 24.3%, and interestingly, non-Hispanic black/non-Hispanic white males represent only 2.0% (Table 3).

According to the American Cancer Society’s Cancer Facts & Figures (2021), 10,590 people are expected to die of cancer in 2021. In addition, the American Cancer Society (2021) underscores that approximately 4020 people will be diagnosed with prostate cancer in Alabama, and 480 people are expected to die, which makes prostate deaths number five out of the top ten cancer death in Alabama. Data also show that Alabama’s prostate cancer mortality rate is 22.9, significantly higher than the national rate of 19.7 (American Cancer Society (2021)). Among the mortality rate, black males in Alabama have a considerably higher prostate cancer mortality rate than white males, with a rate of 47.1 versus 18.1. In addition, Table 4 represents the age relationship with prostate cancer screening in Alabama. Two sets of data (Alabama and the U.S.) are presented in Table 4. The age between 50–59 and 65 and above for prostate cancer screening in Alabama’s male population is much higher than in the U.S. It underscores the severity of the prostate cancer epidemic in Alabama. Although the data mentioned above is higher, Alabama’s white and black prostate cancer screening rates are much lower than the national level (41.6 to 43.9 and 30.7 to 33.4, respectively). It is a compelling healthcare and policy issue to comprehend that the prostate cancer rate in Alabama is significantly higher than the national rate. However, the screening rate does not indicate a higher rate than the national rate (Table 4). This conveys various healthcare policy formulations, such as:

1. Alabama’s prostate cancer population lacks awareness of the cancer health risk
2. Lack of willingness for cancer screening
3. Lack of access to cancer healthcare facilities
4. Lack of data gathering or data distribution on prostate cancer

**Table 4:** Percentage of Prostate Cancer Screening, Men 50 and Older, Alabama and the U.S., 2018. Source: Behavioral Risk Factor Surveillance System, Centers for Disease Control and Prevention (2021) [54].

PSA within the Past Two Years (2021–2022)	Alabama	United States
50–59 Years Old	39.9	26.6
60–64 Years Old	37.9	40.4
65 Years and Older	60.9	46.5
White	41.6	43.9
Black	30.7	33.4
Low Education	26.8	22.9

To understand the association between diabetes and prostate cancer, we used data from the Alabama Department of Health (2013) and developed GIS maps (Figure 4C). The GIS analysis illustrates that a large percentage of the Black Belt region’s male population is diabetic, which correlates stronger with the prostate cancer epidemic than in non-Black Belt counties. The following counties’ health comparison between two distinct situations (diabetes/prostate) are outlined. Counties such as Lowndes (108/180), Montgomery (91/148), Crenshaw (101/116), Bullock (106/139), and Russell (125/100) demonstrate a similar pattern. It is also noted that there are a few neighboring countries in the Black Belt, such as Monroe (106/110), Conecuh (115/81), and Washington (95/104), which also have a close relationship between diabetes and prostate cancer. These data demonstrate that the majority of non-Black Belt counties indicate fewer diabetes cases and lower prostate prevalence, such as Talladega (42/99), Cleburne (25/94), Jackson (52/89), Baldwin (57/89), and Lauderdale (50/78), which suggests that minority communities are struggling with numerous health disparities at once. It illustrates the more significant effects of economic oppression on health as a whole, as it is not just one epidemic that stems from the results.

### 3.5. Obesity, Prostate Cancer, and SES

Obesity is one of the most potent dietary/lifestyle factors correlated with prostate cancer [55]. Prior data have shown that obese men have a greater risk of developing prostate cancer. Another study reported that an extended period of weight gain could increase the prostate size, reducing standard biopsies’ ability to detect cancer at an earlier stage [56,57]. Recent data have identified the relationship between obesity and prostate cancer, suggesting those obese populations are more vulnerable to prostate cancer risk [58,59,60].

The spatial pattern of male obese percentage and prostate cancer per 100,000 people is shown in Figure 4D. The GIS analysis found that most counties’ obesity conditions in Alabama were between 40 and 44 percent. A few counties had less than 40 percent and more than 35 percent. A handful counties out of 67 in the state are shown in the GIS with less than 35 percent, such as Baldwin, Limestone, Madison, and Shelby, which are economically and socially better off counties [25]. 

A strong correlation was observed between obesity, prostate, and poverty in the Black Belt region. Out of 17 Black Belt counties, only two counties—Montgomery and Pike—had less than 40 percent (37% and 39%, respectively). In comparison, all other 15 counties represented an adult obesity rate of 40 percent or higher due to poverty and socioeconomic factors. It has been acknowledged that Alabama is the fifth most impoverished state in the nation, and individuals who live in the Black Belt counties lack access to fresh food, affordability for quality food, and a lack of healthcare programs that spread awareness on dietary health [24]. Further, a lack of spatial distribution of resources among impoverished counties due to people’s SES highly influences prostate cancer incidences, including other diseases. 

**Figure 5:**
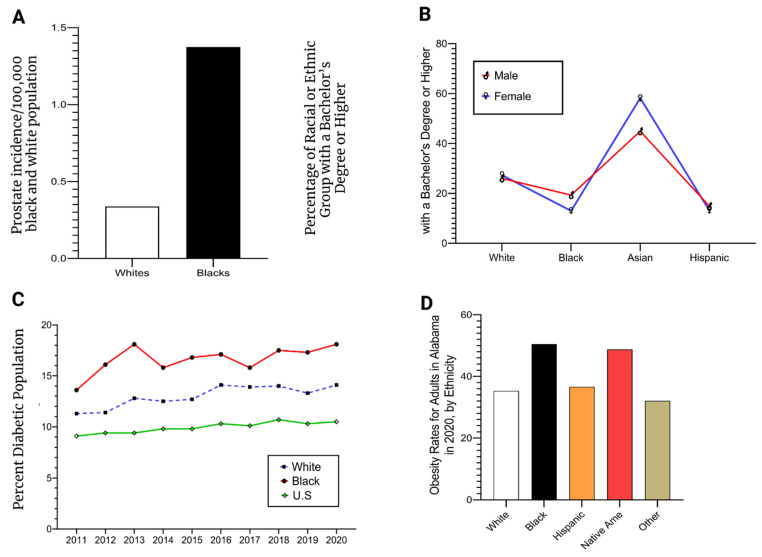
Four factors that cause prostate cancer health disparity in Alabama. (**A**) represents the prostate cancer rate (%) for Alabama’s white and African American male population. The county-level prostate and population (for white and African American males) data were separately aggregated and divided by 100% to obtain the percentage. Although African American is only 27 percent of the state population, African American males face a considerable spike in prostate cancer compared to the white male population. Similarly, (**B**) displays the 4-year college-level degree achievement, (**C**) illustrates the diabetes epidemic, and (**D**) depicts the obesity status in Alabama. Data source: 5A-Prostate Cancer Incidence, Black and White Male Population; American Cancer Society (2019), accessed on 25 December 2021; 5B-Percentage of Racial or Ethnic Group with a Bachelor Degree or Higher; U.S. Department of Census (2018), accessed on 25 December 2021; Percentage Diabetes Population; Center for Disease Control, Behavioral Risk Factor Surveillance System, (2020), accessed on 25 December 2021; and Percentage Obesity Rates for Adult in Alabama; www.statista.com, (2020) [2,24,25,54,61], accessed on 25 December 2021.

All four health and SES factors (prostate, education, obesity, and diabetes) were mapped using a statistical breakdown to understand Alabama’s overall health disparity status. It is stunningly observable in every situation that the African American population holds higher cases than other communities. It is also clear that the lack of availability and affordability of health and socioeconomic factors impact African American communities’ health conditions. Figure 5A,D illustrate four different scenarios for both SES and health conditions in Alabama. Education status is a vital factor that influences health care states. Data was gathered from the US Department of Census (2018) to comprehend the racial or ethnic groups’ education status with a bachelor’s degree or Higher (Figure 5). According to Figure 5B, Black men’s and women’s bachelor’s degree or Higher education holders are way below compared to Asian and White. 

Also, we examined the percentage of the diabetes population as pre-existing conditions that could lead to prostate cancer. Data was compiled from the Centers for Disease Control and Prevention and Behavioral Risk Factor Surveillance System (2020). Data illustrates that the diabetes epidemic condition is far higher among African American males than among white or US populations. The prevailing socioeconomic and health disparity situations are crucial to comprehend the prostate cancer health condition of people in Alabama as they closely correlate. It is profoundly significant among minority populations because they have been systematically marginalized from the mainstream for decades.

## 4. Overall Strengths and Limitations

Using the spatial analysis concept (GIS) within the prostate cancer epidemic in Alabama is a novel idea to comprehend the distribution of health care, access to facilities, and, most importantly, integration of the distribution of socioeconomic status and disparity. Better healthcare policy planning requires a series of data formulation: (1) spatial distribution of healthcare systems and availability of professionals, (2) correlation between cancer and other health epidemics, (3) demographic patterns, (4) infrastructure facilities, (5) socioeconomic status, (6) environment conditions, and, most importantly, (7) health awareness programs. Since GIS can pull to gather all data sources into one spatial database and represent diverse data layers on multimedia platforms, policy planners have the opportunity to witness them from multiple perspectives on the relationship between the cancer epidemic and SES. 

There are a few limitations that we encountered in this research. The research purely represents the outcome using secondary data sources. It would be excellent if we had opportunities to include individual interviews from major ethnic (white and black) sources to validate the data. However, we could not perform the interview due to COVID-19 restrictions. In addition, finding uniform secondary data sources to correlate prostate cancer with other health epidemics and SES was challenging due to the lack of available information online. The state cancer registry underlines a general pattern of prostate cancer instead the detail—for example, age, ethnicity, SES, and region. It is also essential to highlight that finding and downloading GIS format (shape file) cancer data are challenging. This could be related to:

1. Lack of GIS expertise in the state-level of cancer registry to perform the spatial database (shape files)
2. Lack of funding
3. Protection of the privacy of health information
4. Lack of awareness of the desire to disseminate prostate cancer information

## 5. Conclusions

**Figure 6:**
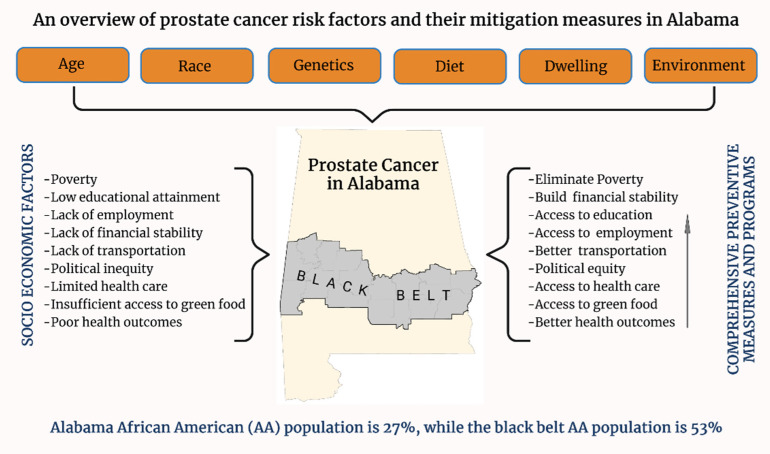
An overview of prostate cancer risk factors in Alabama. This figure summarizes the risk factors and preventive measures that need to be considered to reduce prostate cancer health disparity and bring equity among races.

Alabama is one of the top states concerning the cancer epidemic, and most importantly, prostate cancer is one of the top five health diseases in the state. We examined the impact of SES on prostate cancer in Alabama using Geographic Information Systems. The data showed that SES is critical for prostate cancer incidents in Alabama. SES factors such as poverty, education, income, diabetes, obesity, and access to health care are discussed. Our findings support the statement that the relationship between SES, race, and ethnicity is closely linked [9,18,42,62]. SES, race, and ethnicity generally segregate communities, suggesting that low SES is connected to a significant health disparity based on the pervasive and persistent social status in Alabama’s Black Belt region (Figure 6).

Comprehensive development plans and programs targeting better education and employment opportunities are essential to relieving the low social-economic status of the underserved communities. Effective policy interventions must address some impediments in reducing prostate cases and mortality. We hope that prostate cancer education among African American men in the Black Belt region can create a positive outcome. Policymakers have a significant role in allocating resources to improve access to screening, education, and awareness interventions. To reduce prostate cancer disparity, robust screening guidelines may be needed. It is also imperative to have effective educational programs on prostate cancer to raise awareness among African American men. Outreach programs can be launched through health facilities focusing on prostate cancer; new technology-based facilities in the region are vital to increasing knowledge and screening. 

Prostate cancer cases and mortality among African American men in the Black Belt region lingered for decades [29], which requires favorable decisions to tackle low social-economic status and health disparity. In conclusion, these findings are helpful to policymakers to understand existing challenges faced by the underrepresented communities and make better decisions by recognizing where and how to formulate policies to improve the current conditions. In summary, dedicated research and proactive policy have the chance to save thousands of lives, but the initiative is paramount. Marginalized communities, such as the African American communities within the Black Belt, have been fighting this upward battle against health inequity for generations, lacking the information and resources that save their more affluent peers. Therefore, addressing the socioeconomic challenge is the first step in making accessible health care a right.

## Figures and Tables

**Table 1:** Alabama and the United States prostate cancer incidence rates by race.

Category	Alabama	Unites States
All Races	119.4	104.7
White	97.4	95.4
Black	185.7	169.5

Source: Alabama Statewide Cancer Registry, 2018.

**Table 2:** Age-adjusted percentage of persons 18 years of age and over with diabetes, 2018.

	Non-Hispanic Black	Non-Hispanic White	Non-Hispanic Black/Non-Hispanic White Ratio
Men	13.4	8.7	1.5
Women	12.7	7.5	1.7
Total	13.0	8.0	1.6

Data source: CDC 2021 [54].

**Table 3:** Diabetes death rates.

Age-Adjusted Diabetes Death Rates per 100,000 (2018)			
	Non-Hispanic Black	Non-Hispanic White	Non-Hispanic Black/Non-Hispanic White Ratio
Male	47.6	24.3	1.95
Female	33.1	14.3	2.31
Total	39.3	18.9	2.07

Data source: CDC, National Vital Statistics Report, 2021 [54].

## Data Availability

Data reported in this article is publicly available and has been referenced.
